# Evaluation of the Functionality and Effectiveness of the CORE Group Polio Project’s Community-Based Acute Flaccid Paralysis Surveillance System in South Sudan

**DOI:** 10.4269/ajtmh.19-0120

**Published:** 2019-10

**Authors:** Anthony Kisanga, Bausumo Abiuda, Peter Walyaula, Lee Losey, Omongot Samson

**Affiliations:** 1 CORE Group Polio Project/South Sudan, Juba, South Sudan;; 2 HIGH PSL 9114 LTD., Juba, South Sudan;; 3 CORE Group Polio Project, Washington, District of Columbia

## Abstract

This article describes the functionality and effectiveness of a community-based acute flaccid paralysis (AFP) surveillance system designed and implemented by the CORE Group Polio Project (CGPP) in conflict-affected and inaccessible areas of South Sudan between October 2015 and September 2017. The findings are based on interviews with key informants and focus group discussions as well as data from the CGPP and the management information system of the WHO. Through the implementing partners, the CGPP identified and built the capacity of the community-based surveillance (CBS) system, a system consisting of county supervisors, payam (sub-county) assistants, and community key informants. This structure played a critical role in the identification and reporting of AFP cases. The CGPP also established partnerships with other key players–local and international–to reach greater numbers of people, particularly displaced populations. Evaluation findings show an increase from 0.0% to 56.4% of cases reported through the CBS system between January 2016 and June 2017, and 80.0% of the cases reported within WHO standards of 24–48 hours were through the CBS system, whereas 20.0% were through the facility-based system. The CBS system also recorded an increase from 36.0% in 2014 to 92.0% in December 2016 for the number of counties that were reporting AFP. A CBS system is, therefore, a valuable complement to facility-based surveillance in insecure environments or where the population has limited access to facilities. Community-based surveillance systems also have the potential to identify cases of other infectious diseases of public health importance.

## INTRODUCTION

Civil war broke out in South Sudan in 2013 just 2 years after the country gained independence, leading to the internal displacement of 1.9 million people. More than two million people sought refuge in neighboring countries.^1^ Many health facilities were destroyed and a large portion of the health facility staff members were displaced or killed leading to a collapse of the health system, including immunization and surveillance functions.

Jonglei, Upper Nile, and Unity State were among the most affected states. The non-polio acute flaccid paralysis (NPAFP) rate per 100,000 children younger than 15 years as recorded by the surveillance system declined considerably between 2013 and 2014: from 3.3 to 1.0 in Jonglei; 1.7 to 1.4 in Unity State; and 3.5 to 1.3 in Upper Nile–all substantially lower than the expected rate of at least two cases per 100,000 children.^2,3^ In addition, there were several silent counties and subcounties that reported no cases at all. South Sudan experienced an outbreak of circulating vaccine-derived poliovirus (cVDPV) in Unity State in 2014 and 2015.

The Horn of Africa Technical Advisory Group concluded that the AFP surveillance system at the subnational level in South Sudan was not sensitive enough to detect low levels of ongoing wild poliovirus (WPV) transmission. A surveillance review conducted in South Sudan by external reviewers in 2011 indicated that AFP surveillance was only sensitive in areas that were accessible to the Polio Eradication Initiative field staff. The review revealed that clear gaps existed in inaccessible areas as a result of insecurity, isolated geographical locations because of flooding, and unusable roads. The WHO reported in 2016 that although South Sudan had been polio-free since 2009, it was still at risk of WPV transmission because of inadequate AFP surveillance, low immunization coverage, insecurity, and population movement.^4^

### Polio situation in South Sudan.

Although the last indigenous WPV case was reported in 2001, South Sudan experienced large imported WPV outbreaks in 2004, 2005, 2008, and 2009, affecting nine of the 10 states. The last case was reported in June 2009.^5^ In 2010, the Global Polio Eradication Initiative categorized South Sudan as one of the four countries in Africa with re-established WPV transmission.^6^ In addition, South Sudan experienced repeated outbreaks of cVDPV in Warrap and Western Equatoria States in 2011 and 2012. In 2014, South Sudan reported another outbreak of cVDPV type 2 (cVDPV2) in the Protection of Civilian Sites^1^ and in Bentiu, Rubkona County, Unity State. An additional case of cVDPV2 was reported in June 2015 from Mayom County, Unity State. A detailed investigation of this case showed that the child had no history of polio vaccination (classified as a “zero dose child”) and the community had not been reached with routine immunization or supplementary immunization activities since December 2013.

### The surveillance system for polio in South Sudan.

Before the collapse of the health system, AFP surveillance in South Sudan consisted only of a standard facility-based system, meaning that cases of AFP were reported by health facilities when patients with compatible symptoms presented for treatment.^2^ The system was initiated in South Sudan in 1998 through Operation Lifeline Sudan,^3^ with support from the WHO and non-governmental organizations (NGOs) in locations that were not under the control of the government.^5^ The surveillance system was primarily implemented as a parallel system using county supervisors to investigate cases and send stool samples for testing. Before the outbreak of the civil war in December 2013, the system was performing well, with an NPAFP rate of more than two per 100,000 children younger than 15 years and a stool sample adequacy rate of more than 80%, both of which met international standards.

### The community-based AFP surveillance system introduced by the CORE Group Polio Project (CGPP) in 2015.

In October 2015, in response to the destruction of the existing surveillance system, the CGPP designed and implemented a community-based surveillance (CBS) system in 34 counties of the most war-affected states of Unity State, Jonglei, and Upper Nile. This was conducted in close collaboration with the WHO, the Ministry of Health, United Nation Children’s Fund (UNICEF), and McKing Consulting with financial support from the Bill & Melinda Gates Foundation and the United States Agency for International Development.

The CORE Group is an association of international NGOs working with communities around the world to help them improve their health. The CGPP is a multicountry, multipartner initiative providing financial support and on-the-ground technical guidance to strengthen host-country efforts to eradicate polio.^7^ A U.S.-based global secretariat provides overall technical assistance and financial management to coordinate collaboration among partners. A national CGPP secretariat composed of a small team of technical advisors facilitates communication, coordination, and transparent decision-making among all partners and provides funds for the international and national/local NGOs to support the field work as directed by the Global Polio Eradication Initiative.^7^ From its inception in 1999, the CGPP has developed a unique model of collaboration and coordination that transforms NGO contributions from small, isolated activities into large-scale collaborative interventions that optimize the intense, community-based programming of NGOs with the large-scale coordinated efforts needed to achieve polio eradication.

The concept and design of the CBS system in South Sudan was both simple and innovative, building on CGPP Ethiopia’s use of community mobilizers to identify suspected cases of AFP. The CBS system in South Sudan responds to the need to identify cases of AFP in an environment without a formal health infrastructure to provide basic health services and a lack of security and transportation for external surveillance staff to adequately support surveillance work. The CBS system is based on the assumption that the relatively rare event of acute onset paralysis in a child will elicit some form of health-seeking behavior, and the general nature of small communities will ensure that this rare event becomes known throughout the community and, in particular, to various influential individuals. In the absence of a formal health facility, a mother will take her child to a traditional healer, a religious leader, an informal pharmacist working in the local market, a traditional birth attendant, a community leader, or an elder seeking advice, prayers, and assistance. In addition, bad news tends to spread just like a virus, repeated from person to person. The CBS system builds on these assumptions to complement the formal facility-based surveillance network of health facility–reporting sites with a network of community-based informants who in the course of their daily lives are likely to come into contact with or hear about a case of AFP in their surroundings. The informants are chosen from among these people.

The CBS system does not expect the informants to actively work as surveillance personnel, and they are not paid. In fact, AFP is rare enough that most informants will never come across a case, but when they do, they should recognize it, remember the training they were given, and know who to inform. The concept and design are simple, but of course, the devil is still in the details of implementation and oversight in a war-ravaged insecure country. In this context, we worked with yet another assumption, which was that whereas international NGO and United Nations staff would not be able to travel to the insecure areas of the country, national NGOs could hire and train local staff already in these areas to train and supervise the community-based informants. Hiring locally based workers through local NGOs meant that none of the hired staff were being subjected to any new risks beyond what they were already facing.

Based on this concept and in close collaboration with the WHO, the Ministry of Health, UNICEF, and McKing Consultants, the CGPP established a network of community key informants who reported any suspected AFP case. The CGPP worked with three national NGOs: Bio AID (an organization that operates in 16 counties in Jonglei, Eastern Equatoria, and Upper Nile states), Universal Network for Knowledge and Empowerment Agency (which works in seven counties within Upper Nile and Jonglei), and Support for Peace and Education Development Program (which operates in 11 counties, mostly in Unity State, Upper Nile, and Jonglei states). In total, these partners worked in 34 counties within the four states in South Sudan, which make up 43% (34/80) of the total number of counties covered in the country (Table 1).

**Figure 2.**
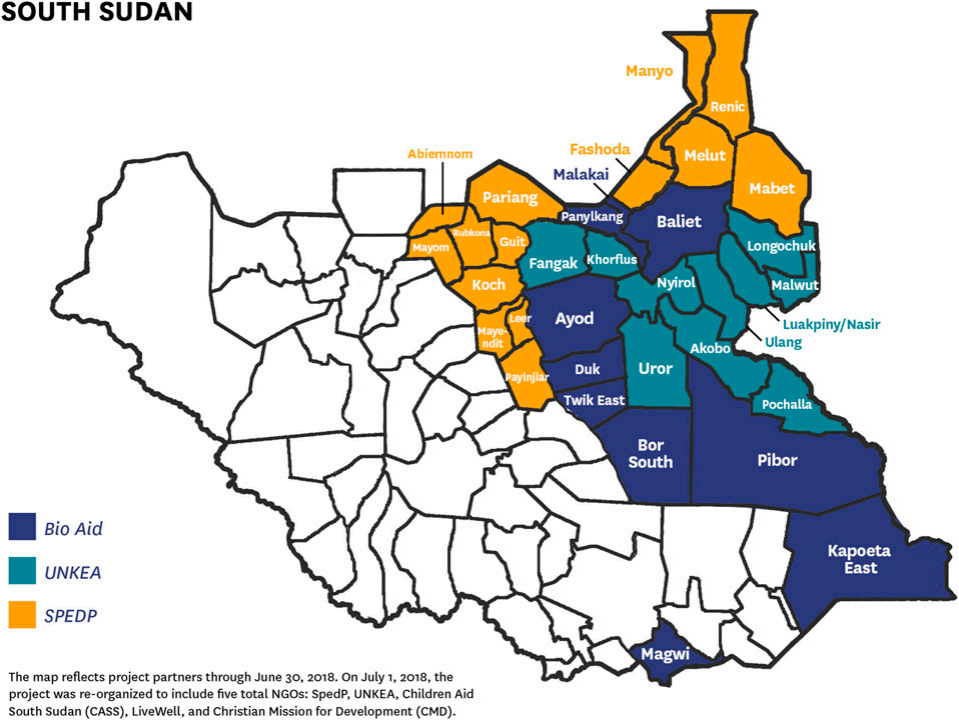
Map of the CORE Group Polio Project community-based surveillance system implementation areas and responsible NGO partners in South Sudan.

**Table 1:** Population of areas where the CORE Group Polio Project community-based surveillance system is operating in South Sudan

State/county	Population under surveillance			
	All ages	< 1 year	0–< 5 years	0–< 15 years
Upper Nile	1,179,984	47,424	248,827	557,424
Jonglei	1,555,989	63,304	327,660	660,593
Unity State	937,510	37,501	196,877	440,628
Kapoeta East (Eastern Equatoria)	30,197	1,887	9,436	18,873
Total	3,703,680	150,116	782,800	1,677,518

### Structure of the CGPP community-based AFP surveillance system.

**Figure 1.**
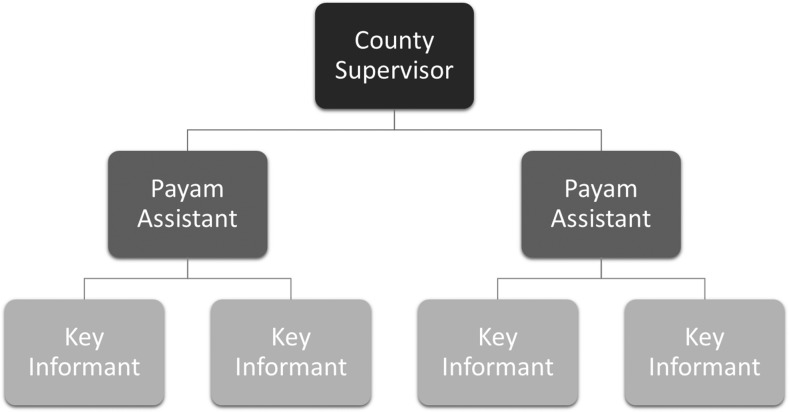
The structure of the CORE Group Polio Project community-based surveillance system at the county level in South Sudan.

The CGPP CBS system has 34 county supervisors who oversee the system at the county level and supervise four to eight payam (subcounty) assistants.^4^ A total of 230 payam assistants were recruited and deployed at the payam level to supervise the activities of 3,228 community key informants, whose task is to detect and report any suspected AFP cases in their villages (Figure 1). The community key informants are mostly illiterate people but respected in the community and are sought out when an adverse event occurs in the community. These community key informants include traditional healers, teachers, church leaders, chiefs, traditional birth attendees, women leaders, and youth leaders. The community key informants are trained on key messages regarding how to identify a suspected AFP case. Each payam assistant supervises a minimum of 15 community key informants and visits each of them at least once a week to see if there is any suspected case reported to the key informants.

In some instances, the community key informants travel to inform the payam assistant, who then travels to the village to verify that the paralysis is acute (of recent onset) or not. If it is an acute paralysis case, he/she fills out the payam assistant portion of the initial reporting form and submits it to the county supervisor within 48 hours. The county supervisor visits the scene of the reported case and investigates further to confirm that the paralysis is not long-standing and documents this on the county supervisor portion of the initial reporting form. The county supervisor also lists all the AFP cases in a simple table, with one line of the table containing all of the information about the case:

1. Name
2. Age in months
3. Location
4. Date of onset of the paralysis
5. Date of notification
6. Date of reporting of the case to community key informants
7. Names of payam assistant and county supervisors
8. Date of the referral to WHO field supervisor for validation
9. Date of investigation
10. Whether it is an acute or chronic case
11. Date of stool sample collection
12. Date that the specimen was transported to the Public Health Laboratory in Juba.

This information helps in tracking the AFP case if the laboratory results confirm the presence of poliovirus in the stool specimen. If it is a new case detected within 72 hours from the onset of the paralysis, it will be reported to WHO field staff to validate the case, initiate stool sample collection, and transport the specimen. In instances where there is no WHO field staff available, the community supervisors for the CGPP CBS system are trained to collect stool samples and transport them to the WHO reference laboratory in Juba.

The CGPP developed standard operating procedures that include a supportive supervision checklist that is used by the county supervisors along with a field activity log book, which documents the activities of payam assistants, including their supervision of community key informants. The county supervisors are supposed to visit every payam assistant under their jurisdiction every month. The payam assistants are expected to visit weekly each of the community key informants who report to them. All these visits are documented and reported monthly to the implementing NGO, which compiles several county reports into one NGO report. All three NGO partners send their updated line lists to the national CGPP secretariat. These line lists include recent cases, old cases, cases from which stool samples have been collected and transported to Juba, and recent cases with no stool sample collected either because of a lack of a cold chain to store the sample until it is transported to Juba or because the person with AFP relocated to another area because of insecurity. At the national level, all the data from the 34 counties are compiled into one CGPP database to assess the functionality of the CBS system. Figure 2 shows the CBS system implementation areas and the responsible NGO partners.

### Measurement of the quality of the CBS system.

The quality of the CGPP CBS system in South Sudan was measured by two internationally accepted indicators: 1) the NPAFP rate (the number of NPAFP cases per 100,000 children aged 15 years or younger per year) and 2) the percentage of cases of AFP in which the stool sample is adequate. Adequacy is defined as the collection of two stool specimens 24–48 hours apart within 14 days of onset of paralysis. An NPAFP rate of ≥ 2 per 100,000 children aged 15 years or less, and a stool adequacy rate of ≥ 80% is considered sensitive enough to detect any presence of AFP caused by circulating poliovirus. The quality of the CBS system is also measured by how many AFP cases are reported within 24–48 hours from time of onset of paralysis and investigated within 48 hours from notification. The number or percentage of silent counties is an additional measure of the quality of the surveillance system.

### Objectives of the evaluation.

The main objective of this evaluation is to document the contribution of the CGPP CBS system to improve the functionality, sensitivity, and effectiveness of AFP surveillance, with attention focused on the conflict-affected and hard-to-reach areas in South Sudan.

We analyzed data collected by community key informants over a period of 18 months from January 1, 2016, to June 30, 2017. In addition, we carried out special field visits to six of the 34 counties where the CGPP had implemented the CBS system without interruption and where security was sufficient to enable a field visit. Nine counties in Unity State were excluded because at the time of the evaluation, the CGPP CBS system had not been operating there for two full years. In addition, of the 25 counties where the CGPP CBS system started, 13 counties were insecure at the time of the evaluation, so field visits were not possible at the time of this evaluation. Twelve were secure and had implemented the CBS system continuously for 2 years without interruption and, thus, were eligible for selection. Of these 12 eligible counties, we randomly selected two counties from Jonglei, three from Upper Nile (because it has more counties), and Kapoeta county from Eastern Equatoria state (the only county in that state where the CBS system was implemented) (Table 2).

**Table 2:** Counties selected for the evaluation

State	County
Eastern Equatoria	Kapoeta East
Jonglei	Akobo
Jonglei	Pibor
Upper Nile	Melut
Upper Nile	Renk
Upper Nile	Maban

The evaluation methodology consisted of a desk review of key CGPP and WHO surveillance documents, reports at the national level, and interviews with key informants at the county level. In addition, interviews and focus group discussions were conducted with relevant partners and stakeholders engaged in the CGPP CBS system (see Table 3). Quantitative data were obtained from routine reports of the CGPP CBS system and the WHO line-list for 2016 and 2017.

**Table 3:** Participants in the qualitative evaluation

Target	Type of qualitative evaluation conducted	Number of participants
County supervisor	KII	6
County health director	KII	6
Payam assistants	FGD	24
Community key informants	FGD	72
UNICEF	KII	1
WHO	KII	1
Ministry of Health	KII	1
Bill & Melinda Gates Foundation Polio Consultant	KII	1
Bio AID	KII	2
Support for Peace and Education Development Program	KII	2
Universal Network for Knowledge and Empowerment Agency	KII	2
Total		118

FGD = focus group discussion; KII = key informant interview.

### Sampling strategy.

Participants for the qualitative study were selected based on whether they had knowledge of the CGPP CBS activities and of the health facility–based surveillance system. Guides were developed for the focus group discussions and for the key informant interviews. Random sampling was used to recruit participants for the focus group discussions. The research team, with the help of county supervisors, randomly selected payam assistants and community key informants to participate in the evaluation. A total of 118 participants were interviewed, of whom 108 were from the six selected counties and 10 were working at the national level in Juba (the capital).

### Data analysis.

During the data analysis, multiple perspectives were included where greater participation was needed to help cross-check accuracy and improve critical reflection, learning, and utilization of information. Stakeholders at all levels were included during the evaluation process to help ensure that evaluation findings were acceptable and credible, ensuring ownership of the findings, conclusions, and recommendations. These included community key informants, county supervisors, payam assistants, county health directors, Bill & Melinda Gates Foundation field staff, Ministry of Health staff from the Expanded Program on Immunization, WHO and UNICEF staff, implementing NGO partner staff, and CGPP secretariat staff based in South Sudan.

We analyzed qualitative data manually using content analysis with coding of themes aligned to a set of categories. We coded and classified content to identify important messages. We grouped all coded content. These qualitative data supplemented the findings obtained through document reviews, from which we extracted quantitative data.

### Ethical considerations.

The interviewers provided participants with information about the evaluation and the purpose of their participation in a language they understood. The interviewers obtained consent from all the participants. The consent procedure conveyed that participation was voluntary and all the information provided by the respondent would remain strictly confidential. Some of the documents included in the review contained sensitive and confidential data about the CGPP CBS system. These documents were kept in a protected location, and any sensitive information, including individual names, was removed.

## RESULTS

### Functionality of the CBS system.

Document review indicated that the CBS system exists according to the submitted reports. Minutes were available of monthly meetings at the CGPP secretariat for 2 years during the study period. We reviewed these to assess the progress of the CBS system, challenges encountered, and recommended actions. Field visits to six counties confirmed the presence of CBS personnel in the form of a county supervisor, payam assistants, and community key informants. We interviewed six county supervisors, 24 payam assistants, and 72 community key informants to gauge whether they knew their roles and responsibilities. The county supervisors reported something similar to that reported by one of them, quoted in the following paragraphs.

The role of county supervisor is to make sure activities are done on a timely basis. I meet with the county health director, payam assistants and the Ministry of Health. I compile a monthly report and monthly financial report; I plan and supervise all payam assistant activities. We select community key informants and then we train them in coordination with WHO guidelines. The county supervisors develop forms to make sure that the information for the key informants and payam assistants is fully recorded, and I make sure that all files are well-kept. I recruit new key informants and payam assistants when resignations occur.

The payam assistants who participated in the focus group discussions indicated that they understand their roles and responsibilities as far as CBS is concerned. Examples are as follows.

We meet with and provide training for key informants, women’s groups, community leader groups, schools, mosques, churches leaders, and others, informing them about AFP surveillance and what kind of symptoms to look for. We also carry out routine visits to the key informants to see how they are coming along with their work and if there have been any cases reported. We also visit the bomas [sub-districts] and villages where AFP cases have been reported.

We give our phone numbers to the community leaders, churches, school teachers, mosques, women’s groups, and of course our staff in the field. Key informants have our phone numbers and inform us immediately when they identify a potential case. Then we go and verify the case that was reported to us and determine whether it is a new or old case. If it is new, we forward the information about the case to our county supervisor.

In Pibor county, community key informants were also interviewed on their roles and responsibilities. Typical responses were as follows.

We search for children who have AFP and report them to payam assistants or county supervisors. The payam assistant comes to evaluate the case and confirms the case.

We conduct social mobilizations to inform the community to take their young children for immunizations.

As a church leader, I tell my congregation during church service about AFP and the need to report any suspected child of AFP.

We talk to the teachers so that they can tell their children about AFP. But also, in our group of 15 community key informants within the payam, some of us are teachers who also raise awareness among our own students.

Sometimes the parents come directly to us if they hear in the area there is a child with AFP. In such cases we visit the child and then, if appropriate, refer the case to a payam assistant.

Key informant interviews both in the field and at the national (Juba) level indicated that the CBS system functions better than the health facility surveillance system. In response to questions about whether the CBS system is functioning, key informants made the following statements.

We monitor the workers employed by CGPP and by WHO. Our findings indicate that the CGPP community-based surveillance staff down to the key informant are actively working as compared to WHO staff who at times are not regular in the field. (national-level expert)

Before, cases at the community level could not be reported unless someone took the child to a health facility. But now, with the community-based surveillance system, it is easy to detect such cases. (county health director)

Because the community has been sensitized about AFP case detection through trainings, the rate of case detection has increased. (county health director)

### Effectiveness of the CBS system.

To assess whether the CBS system is effective, the evaluation looked into three important aspects of surveillance. 1) Did the number of AFP cases reported increase with time (i.e., did the sensitivity increase)? 2) Was the reporting of AFP cases timely? 3) Was the follow-up investigation of reported cases of AFP cases timely? The following are the findings of this aspect of the evaluation.

#### Number of reported cases of AFP.

There was a notable increase in the number of suspected AFP cases reported by community key informants after the establishment of the CBS system in October 2015. Table 4 compares the performance of the CBS system with that of the facility-based system for two time periods: the 12-month period from January 1, 2016, to December 31, 2016, and the 6-month period from January 1, 2017, to June 30, 2017. During the first period, the number of cases reported through the CBS system was similar to the number reported through the facility-based system, but during the second period, the average number of cases per month tripled and was more than five times the number from the facility-based system. There was a similar trend for the number of confirmed cases. The percentage of confirmed cases for which stool specimens were collected was consistently less in the CBS but did improve during the two time periods (from 51.3 to 63.3%).

**Table 4:** Performance of the community-based and facility-based surveillance systems for AFP for the 12-month period from January to December 2016 and the 6-month period from January to June 2017 in the CORE Group Polio Project catchment areas

Type of surveillance system	January 1–December 31, 2016		January 1–June 30, 2017	
	Number	Average per month	Number	Average per month
Cases reported				
Community-based	52	4.3	75	12.5
Facility-based	44	3,7	13	2.2
Total	96	8.0	88	14.7
Cases confirmed				
Community-based	39	3.3	49	8.2
Facility-based	44	3.7	13	2.2
Total	83	7.0	62	10.3

Type of surveillance system	Number	Percentage of confirmed cases for which stool specimens collected	Number	Percentage of confirmed cases for which stool specimens collected
Stool specimens collected				
Community-based	20	51.3 (20/39)	31	63.3 (31/49)
Facility-based	44	100.0 (44/44)	13	100.0 (13/13)
Total	64	77.0 (64/83)	44	71.0 (44/62)
Adequacy of stool specimens				
Community-based	19	95.0 (19/20)	31	100.0 (31/31)
Facility-based	42	95.0 (42/44)	13	100.0 (31/31)
Total	61	95.0 (61/64)	44	100.0 (44/44)

AFP = acute flaccid paralysis.

Source: WHO South Sudan weekly surveillance update and Core Group community-based surveillance AFP line list 2016/2017.

The low stool sample collection rate can be attributed to the fact that many of the children with symptoms of AFP often relocate with their families to safer areas where they could not be traced. Other complicating factors include the cancellation of United Nations Humanitarian Air Service flights (which transport the samples from the field to Juba) and the lack of cold chain equipment to store and transport the samples. Most areas with functional health facilities, on the other hand, are relatively secure, making it easier to collect and transport the samples.

Among the 105 confirmed AFP cases with adequate stool samples collected from both the CBS system and facility-based surveillance system and transported to the laboratory for further analysis between January 1, 2016, and June 30, 2017, none contained identifiable poliovirus. However, this does not rule out the possibility of undetected circulation of WPV in South Sudan because of the ongoing conflict, porous borders, and the presence of an inaccessible large pool of unvaccinated or under-vaccinated children.

The National Ministry of Health Director for the Expanded Program on Immunization in South Sudan was interviewed on the effectiveness of community-based versus facility-based surveillance. Included among his comments were the following.

Facility AFP surveillance is there, but because the health system is not functioning in most places, community-based surveillance under the CGPP has been effective. Community-based surveillance is present in many locations, thus taking services near the people. Where the facility surveillance is working, the CORE Group team on the ground works together with the facility and they report daily.

One community key informant reported the following.

The CORE Group Polio Project has contributed to the improvement of our surveillance system. Honestly speaking, 80% of the AFP cases have been reported by the community key informants. The CORE Group Polio Project has helped our county a lot and we want them to keep on helping us to eradicate polio and also support us in other health-related activities.

A payam assistant reported the following.

Before the project [CGPP] started, people did not know what polio was. They were not aware of vaccination [against polio]. Whenever children had weakness in their legs or arms, their parents would take them to traditional healers. The traditional healers would boil herbs in pots, and these herbs would be directly pressed and squeezed on the paralyzed parts in the legs or arms. This kind of treatment would relieve the sick child a bit, but the paralysis would not disappear. When the project [CGPP] began its activities, people were not fully responding. However, as time went on the community key informants and respected leaders such as traditional healers, traditional birth attendants, women leaders, chiefs, and church leaders in the communities were mobilized and educated on polio, how the disease was transmitted, and how it could be prevented. So, these respected persons were then encouraged to pass these messages to the people. For me, the shift from traditional herbal medicines to vaccination was a very tremendous change. The number of children who were referred to the health facility increased, many children were vaccinated, and the AFP case detection rate increased.

#### Timely reporting of AFP cases.

**Figure 3.**
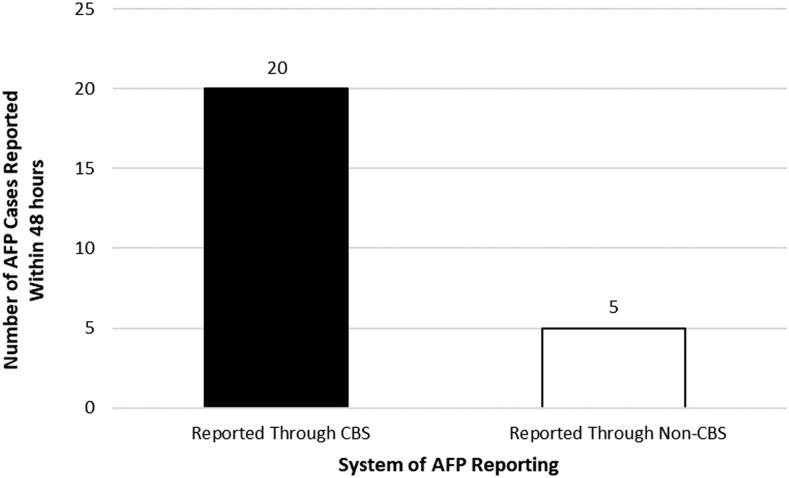
Number of cases of acute flaccid paralysis (AFP) reported within 48 hours of onset, from CORE Group Polio Project community-based and facility-based surveillance system within CORE Group Polio Project catchment areas from January 1, 2017 to June 30, 2017.

Early detection and reporting of an AFP case is one of the most important aspects of an effective CBS system. To assess the timeliness of AFP case reporting from the communities, cases from the CGPP catchment areas were identified both through the facility-based reporting system and through the community-based reporting system. During the 6-month period from January 1, 2017 to June 30, 2017, there were 25 cases that were reported within 48 hours. Twenty (80.0%) of these cases were reported through the CBS system compared with five (20.0%) through the facility-based system as shown in Figure 3.

Representative comments made by community key informants in focus group discussions were as follows.

There was quick reporting of AFP cases from one level to another once a community member notified a community key informant. The quick flow of information from the community key informant to the payam assistant and then to the county supervisor has made the community-based surveillance system smooth, thus enabling surveillance in the community to be more effective than the facility-based surveillance that normally receives cases brought to it when the family has exhausted all options of treatment in the village, including traditional healers. In our [community-based] system we do not delay once a case is identified. We report immediately, and the payam assistants come to verify.

We report immediately any case that we have come across. We are motivated when the county supervisors and the WHO field officers come to the field to investigate the cases we have reported. It shows to the community that we are concerned about our children’s and our community’s health.

#### Timely investigation of cases of AFP.

**Figure 4.**
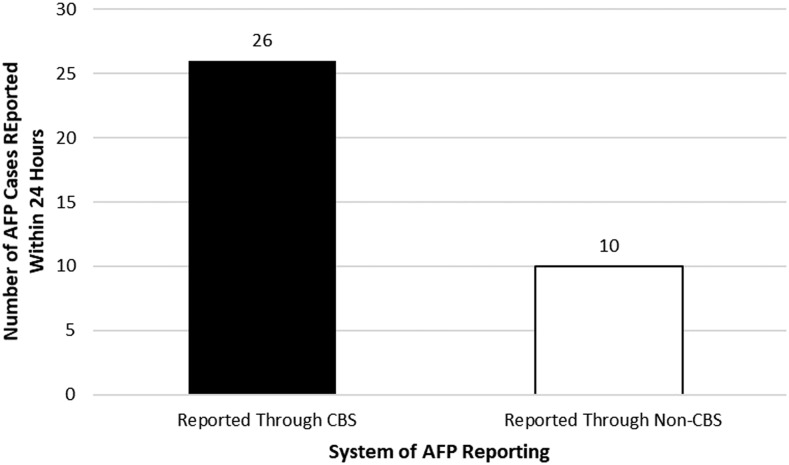
Number of cases of acute flaccid paralysis (AFP) investigated by WHO Field Supervisors and CORE Group Polio Project (CGPP) County Supervisors within 48 hours from notification of AFP case to onset of paralysis within CGPP catchment areas from January 1, 2017 to June 30, 2017.

A review of WHO line-list data from January 1, 2017 to June 30, 2017, showed that of the 44 AFP cases reported in the states of Unity, Upper Nile, Jonglei, and Kapoeta East in Eastern Equatoria from both the CBS and facility-based reporting sites, 36 cases were investigated within the first 48 hours following receipt of notification of the case. Of these, 26 were through the CGPP CBS system and 10 were from facility-based system as shown in Figure 4.

Several community key informants said the following.

Before this [community-based surveillance] project came in place, the situation was not good because there was no case detection in the community. There was no investigation of AFP cases. The changes that have taken place include the reporting and investigation of cases. We have been taught how to verify cases. When a suspected AFP case is reported, it will be verified; if it is a recent case, a stool sample will be taken and we will wait for laboratory confirmation.

Our payam assistants and county supervisors are close to us, and once they are informed of any case, they rush immediately to investigate. This makes the community happy to know that we are there for them.

We do not have [health] facilities close to our area. The fact that CGPP staff are all over the county and can be called upon immediately when there is a suspected AFP case makes us happy.

#### Counties that reported AFP cases as per WHO standard within the CGPP catchment areas.

**Figure 5.**
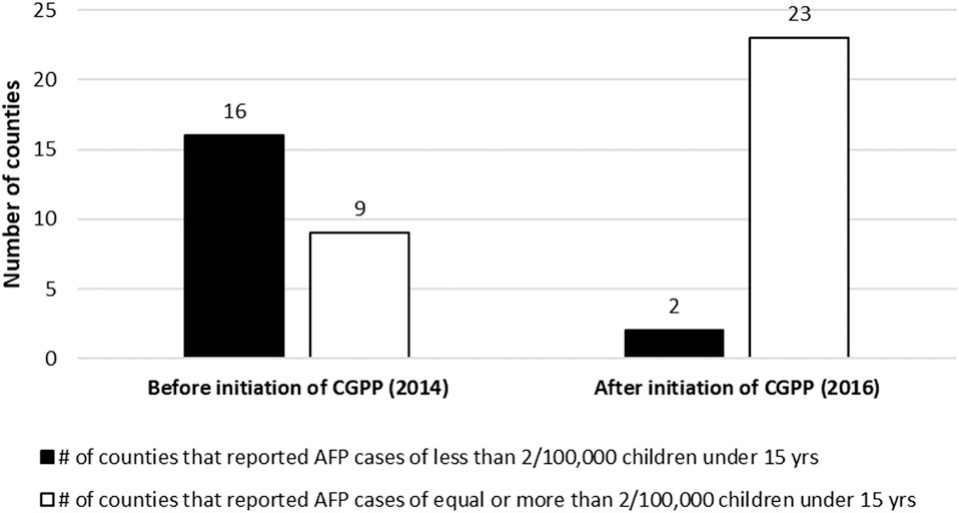
Number of counties within CORE Group Polio Project (CGPP) catchment areas that reported acute flaccid paralysis (AFP) cases according to the WHO standard between January–December 2014 and January–December 2016.

Figure 5 shows an analysis of data from WHO line-list from January to December 2014 (before the initiation of the CGPP CBS system) and from January to December 2016 (when the CBS system had been fully functioning for more than a year) in 24 counties of Upper Nile and Jonglei states, including Kapoeta East county. Figure 5 shows that in 2014, 64% (16/25) of the counties did not report the minimum number of expected cases of AFP. After initiation of the CGPP’s CBS system, almost all (23/25, or 92%) did.

## DISCUSSION

Despite challenging field conditions produced by insecurity, lack of roads, and intermittent flooding, the CGPP CBS system in South Sudan has quickly become highly functional and greatly expands the effectiveness of surveillance that was previously only available through a passive facility-based system in which children with paralysis were brought to a health facility for treatment. Within 24 months after the initiation of the CBS system, 100% of the staff were in place, the staff and volunteers were able to clearly define their roles and responsibilities, trainings were being held, community meetings to sensitize the communities on AFP were being held, clear reporting lines were identified, and active supervision by both the county supervisors and payam assistants was underway. The CBS system monitoring tools were being used to document surveillance activities.

The evaluation also revealed that there had been a notable increase in the number of AFP cases reported through the CBS system following the initiation of the system and an increased percentage of the total AFP cases that had been detected by the community-based system as compared with the facility-based system. Between January 1, 2017 and June 30, 2017, nearly three-quarters of the total AFP cases reported from the geographic areas where the CGPP was operating were obtained from the CBS system compared with only one-quarter from the facility-based system. Furthermore, more than three-quarters of the AFP cases reported through the community-based system during this same 6-month period were reported within the recommended reporting timeframe by community key informants. In addition, analysis of data from 25 counties in the conflict-affected states of Upper Nile, Jonglei, and Kapoeta East county before the initiation of the CBS from January to December 2014 and other data for the period January–December 2016 after the initiation of the CBS system in these CGPP catchment areas indicated that there was substantial increase in the number of counties that reported AFP cases according to WHO standard surveillance indicator of equal to or more than 2/100,000 children younger than aged 15 years in 2016 compared with 2014 before the introduction of the CBS system. Finally, the evaluation revealed that the AFP cases identified through the CBS system had a higher chance of being identified within 48 hours of onset of symptoms than did cases identified through the health facility surveillance system.

Field interviews indicated that factors that contributed to the effective functioning of the CBS system were 1) the presence of a network of community key informants in every village, 2) the presence of supervisory staff who can investigate AFP cases promptly, 3) strong capacity building through training, close supervision, awareness creation, and involvement of the local community, 4) strong coordination, and 5) partnerships. There was a well-established and smoothly functioning structure from the national level down to the sub-district (boma) level, which was able to engage the community in polio eradication that was not possible through the facility-based system. The CGPP is now positioned to use the CBS system for promotion of routine immunization, guinea worm eradication, and control of other communicable diseases such as tuberculosis, kala-azar, measles, and neonatal tetanus as has been performed in other settings.^8^ Such systems could also identify cases of Ebola and pandemic influenza.

Before the initiation of the CGPP CBS in South Sudan in October 2015, there had been 13 silent counties among the 33 (39.4%) that were in the most security-compromised and hard-to-reach areas. Two years after the initiation of the CGPP CBS, only three of these 33 countries (9.1%) remained silent.^9^

In South Sudan, where prolonged civil war has seriously damaged the health services infrastructure and reduced the number of operational peripheral health facilities, the presence of a CBS system is essential. In spite of the inaccessibility of higher-level health personnel to areas of ongoing conflict and the inoperability of health facilities, the CBS system functioned well because of the engagement of the community and because volunteer community key informants were able to identify and report AFP cases.

### Evidence from other studies of the effectiveness of CBS.

Disease surveillance is an important component of any health system. Among other things, if working well, it can quickly detect infectious disease outbreaks that could be detrimental to the health of the community.^10^ In rural Cambodia, a study confirmed that a CBS system can be successful in filling the gaps of the health facility–based surveillance system by rapidly detecting outbreaks, by continuously and effectively monitoring the outbreaks, and by registering vital events.^11^

A study in Ghana^12^ reported the important contribution that CBS volunteers (CBSVs) had made to the eradication of Guinea worm disease (dracunculiasis), rendering the country free of the disease in 2006. According to the study, the CBSVs played a critical role in community mobilization and awareness creation on preventing the spread of the disease, door-to-door administration of drugs, referral of cases to health centers for treatment, and distribution of water filters.

A campaign by the Carter Center on Guinea worm eradication in South Sudan has proven that involvement of community members through the CBS system can be an effective means of eradication of diseases in the community. With 20,582 cases in 2006, South Sudan has been the country with the greatest number of cases, but there were no reported cases in 2017.^13^

An effectively functioning CBS system for AFP is important for geographic areas at high risk of cases of polio—either from importation or from indigenous sources. Community-based surveillance systems can be useful in countries with weak health systems such as South Sudan because with minimal additional effort, a CBS that has been established for polio can be expanded to include other priority communicable diseases. The CBS system for AFP in South Sudan is essential because of its porous borders and the low levels of polio immunization coverage on both sides of the border.^14^ The community engagement required for the CBS system is also useful for strengthening the coverage of other routine immunizations beyond polio and reaching more children through supplemental immunization, both essential components for polio elimination in fragile states.

South Sudan’s CGPP CBS system, implemented in the most inaccessible, insecure, and hard-to-reach areas of the country, is now functioning effectively with a well-developed network of 2,366 male and 1,126 female community informants, payam assistants, and county supervisors.

## CONCLUSION

The CGPP has been able to engage communities in South Sudan to establish a CBS system for AFP that has improved the quality of surveillance greater than what had been previously achieved by the facility-based system. Community-based surveillance has particular relevance for South Sudan (and other conflict-affected or otherwise fragile states at risk of polio transmission) because of the destruction of its health infrastructure and the inability of health staff to work in violent, insecure areas. This same CBS system can also be used to detect other diseases present in South Sudan such as Guinea worm, tuberculosis, kala-azar, measles, and neonatal tetanus and thereby trigger appropriate disease-control responses. Community-based surveillance represents a powerful tool for disease control that merits broader application for disease control and detection of emerging communicable disease threats such as Ebola and pandemic influenza.

## References

[1] UNOCHA, 2018 South Sudan. Available at: https://www.unocha.org/south-sudan/. Accessed February 7, 2019.

[2] World Health Organization, 2014 South Sudan Polio Updates (Weekly Surveillance Update No. 12). Juba, South Sudan: WHO, 22–24.

[3] World Health Organization, 2015 South Sudan Polio Updates (Weekly Surveillance Update No. 15). Juba, South Sudan: WHO, 20–22.

[4] World Health Organization, 2016 South Sudan–GPEI. Available at: http://polioeradication.org/where-we-work/south-sudan/. Accessed February 7, 2019.

[5] WHO EMRO, 2018 WHO EMRO Polio Eradication Initiative South Sudan. Available at: http://www.emro.who.inthb/polio/countries/south-sudan.html. Accessed February 6, 2019.

[6] Wassilak S Orenstein W, 2010 Challenges faced by the global polio eradication initiative. Expert Rev Vaccines 9: 447–449. 20450316 10.1586/erv.10.45

[7] Losey L 2019 The CORE Group Polio Project: an overview of its history and its contributions to the global polio eradication initiative. Am J Trop Med Hyg 101 (Suppl 4): 4–14. 10.4269/ajtmh.18-0916 PMC6776098 31760971

[8] Ratnayake R 2016 Assessment of community event–based surveillance for Ebola virus disease, Sierra Leone, 2015. Emerg Infect Dis 22: 1431–1437. 27434608 10.3201/eid2208.160205 PMC4982166

[9] Stamidis K Bologna L Losey L, 2018 CORE Group Polio Project (CGPP) Final Evaluation Report 2017. Available at: https://coregroup.org/wp-content/uploads/2018/06/CGPP-Evaluation-Report-FINAL-5-10-2018.pdf. Accessed February 7, 2019.

[10] Nsubuga P 2006 Public Health Surveillance: A Tool for Targeting and Monitoring Interventions. Washington, DC: The World Bank and Oxford University Press. 21250345

[11] Oum S Chandramohan D Cairncross S, 2005 Community-based surveillance: a pilot study from rural Cambodia. Trop Med Int Health 10: 689–697. 15960708 10.1111/j.1365-3156.2005.01445.x

[12] Baatiema L Sumah AM Tang PN Ganle JK, 2016 Community health workers in Ghana: the need for greater policy attention. BMJ Glob Health 1: e000141. 10.1136/bmjgh-2016-000141 PMC5321387 28588981

[13] Pallares G, 2018 How South Sudan Stopped Guinea Worm Disease in its Tracks. Available at: https://www.devex.com/news/q-a-how-south-sudan-stopped-guinea-worm-disease-in-its-tracks-92414. Accessed February 4, 2019.

[14] Mbabazi W Lako AK Ngemera D Laku R Yehia M Nshakira N, 2013 Maiden immunization coverage survey in the republic of South Sudan: a cross-sectional study providing baselines for future performance measurement. Pan Afr Med J 16: 110. 24876899 10.11604/pamj.2013.16.110.3164 PMC4033584

